# The Bidirectional in Bilingual: Cognitive, Social and Linguistic Effects of and on Third-Age Language Learning

**DOI:** 10.3390/bs9090098

**Published:** 2019-09-11

**Authors:** Anna Pot, Joanna Porkert, Merel Keijzer

**Affiliations:** 1 Faculty of Humanities, University of Amsterdam, 1012 WX Amsterdam, The Netherlands; a.pot@uva.nl; 2 Department of Applied Linguistics, University of Groningen, 9712EK Groningen, The Netherlands; j.porkert@rug.nl

**Keywords:** bilingualism, aging, third-age language learning

## Abstract

Bilingualism has been put forward as a life experience that, similar to musical training or being physically active, may boost cognitive performance and slow down age-related cognitive decline. In more recent years, bilingualism has come to be acknowledged not as a trait but as a highly individual experience where the context of use strongly modulates any cognitive effect that ensues from it (cf. van den Noort et al., 2019). In addition, modulating factors have been shown to interact in intricate ways (Pot, Keijzer and de Bot, 2018). Adding to the complexity is the fact that control processes linked to bilingualism are bidirectional—just as language control can influence cognitive control, individual differences in cognitive functioning often predict language learning outcomes and control. Indeed, Hartsuiker (2015) posited the need for a better understanding of cognitive control, language control as well as the transfer process between them. In this paper, we aim to shed light on the bidirectional and individual cognitive, social and linguistic factors in relation to bilingualism and second language learning, with a special focus on older adulthood: (1) we first show the intricate clustering of modulating individual factors as deterministic of cognitive outcomes of bilingual experiences at the older end of the lifespan; (2) we then present a meta-study of work in the emergent field of third-age language learning, the results of which are related to lifelong bilingualism; (3) objectives (1) and (2) are then combined to result in a blueprint for future work relating cognitive and social individual differences to bilingual linguistic outcomes and vice versa in the context of third-age language learning.

## 1. Introduction

Research towards healthy aging has attested that engaging in cognitively stimulating experiences—such as playing a musical instrument [1,2], being physically active [3] but also seemingly simple activities such as engaging in meaningful discussions [4]—may promote brain plasticity. This is supported by neuroscientific research that finds the brain to maintain lifelong plasticity through adjusting to such experiences [5,6]. Brain plasticity is said to enhance overall cognitive performance and may also slow down age-related cognitive deterioration. Within this spectrum of cognition-enhancing experiences, bilingualism has taken a prominent and yet complex role.

Bilingualism is a complex cognitive undertaking, requiring individuals to continuously and actively manage two (or more) languages in one mind. This language competition and control has been shown to transfer to enhanced cognitive control more broadly, perhaps most spectacularly manifested as cognitive reserve in older adulthood. Current large-scale studies show that lifelong bilingualism may delay the onset of Alzheimer’s disease and dementia by approximately 4.5–5 years across cultures [7,8,9]. This finding, however, is not robust (see the meta-analysis of Mukadam and colleagues [10]) and research targeting bilingualism’s influences on cognitive reserve with older populations in different regions has produced mixed results [11,12,13].

These inconsistent findings do not imply that bilingualism is not among the cognitively enriching experiences that ‘train’ executive functions to result in cognitive reserve in older adulthood. Rather, they demonstrate that bilingualism is not a ‘trait’ that can be operationalised as cognitive training through competing language systems. Rather, it is a highly individual experience, relating to social contexts in which the languages are used, but that especially in older adulthood also interacts with personality traits and well being levels [14]. Indeed, differences in bilingual experiences in relation to (social) variables are especially prominent at an advanced age [15] and, most compellingly, have been linked to clear demonstrations of differences in brain structure. In older adulthood, lifelong bilinguals show greater white matter integrity in certain brain areas and stronger anterior to posterior functional connectivity [16]. Estanga and colleagues [17] showed that lifelong bilinguals differed from monolinguals even in biological parameters, as they had significantly lower t-tau levels in the cerebrospinal fluid. Bak and Robertson [18] linked this finding to sustained activation of noradrenergic signalling pathways and related it to the late onset of dementia.

The main aim of this study is twofold. It first reviews the work that has adopted an individual differences perspective on bilingualism, focusing specifically on older adulthood (operationalised here as 65 years and older), see Section 2. Building on this, the paper explores the recent work on third-age language learning, in which bilingualism is newly introduced as a life experience to functionally monolingual older adults. Through a meta-analysis of studies within this emerging field presented in Section 3, we explore what new light this perspective can shed on our understanding of bilingualism as a cognitively enriching experience. Indeed, many of the differences in bilingual experiences modulating cognitive outcomes (see meta-study by van den Noort and colleagues [19] in this volume) can be kept under control by introducing a second language proactively rather than examine the effects of lifelong bilingualism retrospectively. Not all individuals pick up a new language as easily in older adulthood. Linked to this, a third perspective on individual bilingual experiences is presented in Section 4: just as language control can influence cognitive control, individual differences in cognitive functioning can predict language learning outcomes and control. The paper ends with a future outlook of the emerging field of third-age language learning and the insights into cognitive and language control that can ensue from it.

## 2. Individual Differences in Bilingual Experiences in Older Adulthood

In a review article that appeared earlier in this special issue of Behavioural Sciences, de Bruin [20] argues for more detailed assessments and descriptions of bilingual experiences in order to determine more precisely how bilingualism contributes to cognitive performance. Although we are by now well aware that a bilingual is not the sum of two monolinguals, a myth that has proven more difficult to bust is that all bilinguals are similar [21]. Bilinguals are typically ‘unbalanced’ in their linguistic knowledge. For example, their vocabularies in each language will be strongly tied to the domain in which this language is mostly used. Therefore, they typically know words in one language that they do not know in the other [21]. But they are also different in the age at which they acquired their languages, how actively they now use them, and in their attitudes towards their own bilingualism. As bilingualism is such a multifaceted phenomenon, it is difficult to give a specific definition that encompasses all the bilingual participants discussed in this paper. For the sake of clarity, however, we regard a bilingual as someone who speaks, learns or (at some point) has spoken or learned more than one language. The call for more detailed assessments and descriptors of bilingual individuals is important in research that seeks to understand how bilingualism, as a life-experience, shapes cognition and the brain.

A large-scale meta analysis by Lehtonen and colleagues [22] is one of the latest in a series of articles, reviews and meta analyses to try to make sense of the inconsistent results in studies examining beneficial effects of bilingualism on cognition. Lehtonen and colleagues [22] corroborated the absence of reliable evidence of a bilingual advantage after systematically reviewing studies on six executive domains. In a short review article, Laine and Lehtonen [23] subsequently highlight three problem areas that drive these inconsistencies in results: (1) research designs, which mostly rely on artificial and discretely imposed mono/multilingual categories; (2) measures, which do not clearly specify which underlying construct they tap; and (3) a lack of a clear theory to explain the underlying mechanisms that drive a bilingual cognitive advantage. Laine and Lehtonen [23] conclude with a call for hypothesis-driven research where the focus of attention on various features of individual bilingual experiences may get us closer to the mechanism(s) underlying a bilingual advantage in cognitive functioning. With a more thorough understanding of individual bilingual differences comes more insights as to why given variables predict better cognitive functioning for one specific group of bilingual individuals, without being of importance in another group.

Whereas previously bilingualism was often operationalised as a static state, categorising individuals in mono- or bilingual categories, current research increasingly views bilingualism as a life-experience operating along a continuum [24,25]. Under this view, bilingualism is a dynamic variable that changes over the lifespan and that impacts on and is being influenced by other experiences throughout life. Especially at the older end of the lifespan, cognitively enriching experiences such as playing a musical instrument—and purportedly also bilingualism—have been claimed to lead to special benefits for older adults in terms of slowing down cognitive decline and contributing to cognitive reserve [26]. At the same time, the accumulation of life-experiences in older individuals makes it especially challenging for research to detail the precise role of language in modulating and/or shaping cognition and disentangling these.

### 2.1. Bilingual Language Usage

Cognitive benefits have been observed for those older adults who have high bilingual management demands (keeping their languages at a high level of activation) and with long-term experience managing these demands [27]. Some researchers find a benefit for users of multiple languages under such high-demand conditions—in a population of oldest-old bilinguals (i.e., 75 years of age and older), Reference [28] reported that the number of languages participants spoke was a better predictor of cognitive test score, beyond other demographic variables. In a large study sample of 2812 older adults (aged between 65 and 101), Reference [29] investigated the role of various individual factors on a range of cognitive and psychological abilities. They asked their participants about the different languages they spoke on a daily basis and found that having a command of multiple languages in old age often contributed to enhanced cognition. However, this was not equally present in all participants and was dependent on other cognitively stimulating activities the participants engaged in, their verbal abilities in the languages they spoke and basic processing speed. Similarly, in an attempt to investigate the role of bilingualism on cognition, Reference [13] found their population of more balanced (in terms of proficiency and use) older Dutch-Frisian bilinguals (aged 65 and up) to outperform monolingual age-matched Dutch speakers on measures of executive function. It is thus not so much the number of languages, but the usage intensity of these languages that relate to enhanced cognitive effects. Bilingual language usage, however, is a distinctly individual experience.

### 2.2. Individual Differences and Language Control

A recent article examined a number of experience-based factors (EBFs) in bilingual language use and their relation to brain structure and functional connectivity [30]. Although not directly targeting older adults (the 65 participants ranged in age from 18 to 52), the research is interesting in its attempt to link functional brain connectivity to bilingual experiences. The researchers targeted a range of linguistic experiences in a diversely bilingual group by means of a questionnaire that documents language use over time in a range of social settings (e.g., home and community settings). Duration of bilingual language use was found to correlate with increased language processing efficiency, a finding they interpreted as signalling more automated grapheme-to-phoneme mapping. Length of L2 immersion (living in the L2 environment) was related to increased automatisation in language control, manifested by adaptations in posterior sections of the right caudate nucleus. The degree of L2 use in social settings positively related to better adaptations in increased demands on language control and selection, visible in the brain through expansions in the left caudate, which is engaged in language switching and selection. Active and sustained L2 use, finally, were found to promote efficiency in language regulation and processing. The results of the study demonstrate that specific EBFs predict specific brain adaptations, highlighting again the need for an individual difference approach to bilingualism.

These findings relating bilingual experiences to brain structure and functional differences are partly in line with earlier research by Prat and Just [31], who investigated brain activity during reading comprehension and related it to vocabulary size of individuals, albeit again not older adults per se. They showed that higher vocabulary scores lead to less brain activity in certain areas, suggesting that the brains of more proficient individuals are more efficient. Moreover, Stocco and Prat [32] note that enhanced brain efficiency leads to greater adaptability when brain tasks become more complex and demanding. Those with more efficient brains adapt better to complex circumstances. Bilingualism might be one such skill that contributes to brain efficiency (Hernandez, 2013). These observations are in line with deLuca et al.’s [30] conclusion regarding increased automatisation of language control, but also with the observation that high bilingual management demands require efficient language processing.

In an overview article on bilingual language processing, Fricke and colleagues [33] argue that even bilinguals of the same proficiency level use a multitude of regulatory strategies to manage their languages. This is influenced by different learning or acquisition experiences and usage patterns, which suggests a plastic view on bilingual language processing, similar to DeLuca et al.’s [30] observations. The fundamental differences observed between individuals in language processing may be an effect of a speaker’s language regulation history. This signifies how an individual adapts his or her language use to the linguistic contexts in which the languages are used and, crucially, were use in the past. This notion lines up with the finding that the interactional context of multilingual language use and experience with this context is imperative to domain-general cognitive performance.

Contextual-dependent findings are at the core of the Adaptive Control Hypothesis [34]. This hypothesis distinguishes three language switching contexts, relating to the intensity and ease of switching: (1) a single language context where one language only is spoken; (2) a dual language context whereby language cues need to be closely monitored in order to select the ‘right’ language; and (3) a dense code-switching context, where both languages are interchangeably used—often even inter-sententially—but no clear monitoring/inhibiting of one language is necessary. Findings on adaptive language control mechanisms that change relative to the language context [35], the observation that a high daily exposure to a bilingual’s different languages better resolves competition between languages [25], and the observations on EBFs and language demands above, all strengthen the evidence that the greatest cognitive benefits are observed in a dual-language context. The continuous adjustment of the control mechanisms could train the brain to become more attentive and efficient in switching between languages. With most of these studies not specifically targeting older brains, it is imperative that this line of work is extended to older adulthood because of reduced brain flexibility that is often noted at this life stage (but see Reference [36]), especially because the contextual bilingual experiences of older adults are more extensive than those of their younger peers.

### 2.3. Individual Differences and The Environment

The context in which languages are used warrants an investigation into the social environment of bilinguals. Indeed, much more than a factor in isolation, bilingualism is a complex, social variable. Language use changes depending on the social domain in which it is used, and is influenced by the degree of switching between languages [37]. Part of DeLuca et al.’s [30] questionnaire tapped L2 use in different environments. Other research looking into the social aspect of bilingualism is starting to emerge (see the chapters in a recently published volume by Sekerina, Spradlin, and Valian, [38]). It is through factors relating to the interaction context of bilinguals—which are inherently distinct—that we can gain a better understanding of and when bilingualism influences cognitive control.

A study by Pot, Keijzer and de Bot [14] examined a large cohort (n = 387) of multilingual seniors (65+) in the northern part of the Netherlands and assessed which aspects of multilingualism and crucially under which circumstances multilingualism could contribute to enhanced cognitive performance. Precisely when considering the usage context of multilingualism, cognitive advantages on a Flanker task were observed. More specifically, individuals who used their different languages in different social domains produced significantly smaller Flanker effect scores, but only when these scores were examined in a cluster with personality characteristics (openness to experience in particular), education and quality of life criteria, observed through partial least squares analyses. Language proficiency and age of onset of acquisition contributed less than the usage of different languages (irrespective of proficiency level and length of language use) in different social contexts. This confirms the observations of earlier work regarding sustained use of different languages in social domains and lines up with the dual-language context of the Adaptive Control Hypothesis. Interestingly, however, is the observation that certain personality characteristics also interacted with cognitive performance, showing bilingualism to be highly intertwined with other lifetime experiences. Openness to experience is in the context of bilingualism perhaps especially enticing.

This is corroborated in a large-scale study towards factors that may influence cognitive performance in older adults. Ihle, Oris, Fagot, Maggiori and Kliegel [39] found that—in a population of 2812 Swiss older adults (65+) from different cantons—the personality variable openness to experience was a significant indicator of better cognitive performance. The authors hypothesize that individuals who score high on the open to experience dimension may have engaged more with cognitively stimulating activities throughout their lives. Indeed, given the interaction between openness to experience and the use of different languages across social domains observed in Pot et al. [14], those individuals with this personality characteristic could be more inclined to seek out more diverse social connections or sustain these, perhaps through taking up a language course or through traveling; all cognitively stimulating activities.

In a later study, Ihle and colleagues [40] investigated the malleability of cognition and found this to be in part influenced by the size of social capital (degree of supportive social relationships and interactions) that individuals accumulated over the lifespan. A large social capital in old age often correlates with enhanced cognitive performance, but also increased well being levels: Ihle et al. identified a link between lower physical and psychological well being levels and lower cognition. Life-experiences can therefore be enriching, but it is difficult to separate the influence of one factor or experience from another and detail its precise contribution to cognition. The interaction of personality characteristics and multilingualism in Pot, Keijzer and de Bot [14] also reflect this.

So far, we have explored how aspects of multilingualism (and connected environmental factors) may promote cognitive functioning at an advanced age. One way to examine the effect of these experiential factors including multilingualism on cognition in old age would be to introduce such activities proactively rather than measure their effects reactively. Previous research on cognitive intervention programmes for older adults have, for instance, explored the effects of music lessons to seniors and demonstrated that, even without lifelong experiences, engaging in such cognitively stimulating tasks promotes cognitive health [41]. Recently, studies of language learning in old age (also termed third-age language learning) have been set up to extend this line of work. Examining the effect of such an activity on an individual could present better insights into the contribution of all these individual factors such as personality, accumulated life experiences, and so forth to cognitive outcomes of bilingualism.

## 3. Language Learning as a Tool to Promote Healthy Aging in Older Adults

Lifelong bilingualism has been demonstrated to reflect differences in brain structure in older adults ([16,17], see above). However, acquiring a new language at a later age too might influence the structures and cognitive abilities of the brain and help to ward off cognitive decline. Schlegel, Rudelson and Tse [6] were among the first to show that the white matter of young adults changes gradually but significantly during an intensive 9-month course of Modern Standard Chinese. Li, Legault, and Litcofsky [36] produced an overview of functional and structural brain changes to follow cognitive training regimes, among which language learning. But these studies targeted younger adults; research on the late acquisition of a new language and its effects on cognition in older adulthood has only very recently emerged. This section outlines and compares eight such studies as part of a meta-study. An inclusion criterion for studies in this review was that actual language training regimes in older adulthood should be implemented other than ideas being posited about what form such language training should take. Three of the included studies focus on the cognitive abilities and types of instruction that predict L2 learning success and rate in older adults, whereas the other five explore the effect of language learning in older adulthood in relation to specific cognitive abilities. By comparing the scant work that has been done in this domain and relate language constellations, teaching methods and intensity to linguistic and cognitive outcomes, a blueprint can be provided for future work in this domain with its ultimate aim to shed more light on the nature of cognitive and language control as well as the transfer between them in the context of bilingualism as a final constituent.

### 3.1. Short Summaries of Research Questions and Aims of the Studies

Before comparing the studies, they are briefly summarized in Table 1 below.

### 3.2. Participants and Group Composition of the Studies

The studies vary considerably with respect to their sample sizes and group setup with the majority of studies (unsurprisingly for an emergent research field) comprising (pilot) feasibility studies with small sample sizes (Mackey and Sachs, 2012 (n = 9); Ware et al., 2017 (n = 14); Kliesch et al., 2018 (n = 10); Pfenninger and Polz, 2018 (n = 12)) [42,43,44,45]. Given the relatively small sample sizes of these studies, their results have a limited generalizability and are exploratative more than anything else. Three of the studies (Bak et al., 2016 (n = 77); Cox, 2017 (n = 43); and Ramos et al., 2017 (n = 43) [46,47,48]) had medium sample sizes and therefore more statistical power. However, Bak et al. [46] compared participants across age groups, which implied that only around one third of the participants were older adults (n = 21). Furthermore, the study of Cox [47] focused on two main variables (explicit instruction and bilingualism), leading to four groups of around 10 subjects per group. The only study with a large sample size was performed by Berggren et al. [49], who tested 160 older adults. It furthermore needs to be pointed out that the definition of third age varied only marginally between the studies, with a mean age of around 68-70 years for most studies (an exception was Ware et al. [43], whose mean age was 75.42 years).

The variability across studies extends to their inclusion of active or passive control groups (of monolinguals). Those studies with a focus on feasibility, language learning rate, or teaching method did not include control groups for the language training condition [42,43,44]. Two studies (Cox, [47]; Pfenninger and Polz, [45]) were interested in late/sequential bilingualism as compared to monolingualism as they set out to reveal possible language aptitude advantages (which Cox [47] indeed found) or cognitive advantages (which was not confirmed by Pfenninger and Polz [45], whose monolinguals outperformed the bilinguals). Cox [47] furthermore investigated whether explicit grammar instructions were helpful to older adults and therefore recruited an implicit and explicit senior condition. Those studies that were interested in language learning as a tool to enhance cognition also included passive control groups [46,48], and/or active control groups but used different means to do so. Bak et al. [46] set off language learning vis-à-vis an English teaching qualification course, an art class, a documentary film course, and a passive control group whereas Berggren et al. [49] included an active relaxation yoga control group. Including active control groups can disentangle the effects induced by language and other cognitively stimulating activities.

### 3.3. Language Constellations

The target languages of the eight studies mainly corresponded to the majority languages of the study locations (with the exception of Mackey and Sachs [42] who focused on Spanish L1 speakers in the USA). Four studies decided to teach English as a target language [42,43,44,45]. While many older adults might be more motivated to acquire English due to its dominant position as global lingua franca, this decision risks prior exposure, which might lead to the course being too simple and not cognitively stimulating enough (cf. Ware et al. [43]). Choosing a typologically more distinct (minority) language, as in the case of Bak et al. [46] and Ramos et al. [48] may be more demanding, leading to more substantial effects, but at the same time runs the risk of being too challenging, with a possible decrease in motivation to learn it as a result (cf. Reference Ramirez-Gomesz [50] on critical foreign language gerontology principles for more details). The same can be said about teaching an ancient language such as Latin, as was done in Cox (2017), motivated by a focus on metalinguistic awareness and language aptitude.

### 3.4. Teaching Methods and Teaching Intensity

The studies mainly employed language courses taught by trained teachers in a classroom-setting with standardized material. An exception was Mackey and Sachs [42], who investigated the effect of personal interaction with trained native speakers in one-on-one sessions based on direct feedback as a method. Two studies opted for technology-based instruction methods and showed that older adults can benefit from using them—Cox [47] and, partially, Ware et al. [43]. While such technological instruction types indeed have certain advantages, such as the opportunity for more self-study if used as part of a blended learning design and increased motivation levels (cf. Ware et al. [43]), there are also certain disadvantages that come with computer-based language learning: some older adults might feel challenged and it may be time-consuming for them to learn how to use technological appliances (Pfenninger and Polz [45] opted, for example, for paper-and-pen cognitive tasks as the computer-based tasks proved too challenging for their seniors).

When it comes to duration and intensity of the training, the language courses of the studies vary considerably. They range from courses that were held once a week for five weeks (Mackey and Sachs [42]), through to a one-week intensive course with daily teaching sessions of four hours (Bak et al. [46]), to eight months of intervention comprising 3.5 h per week (Ramos et al. [48]). The choice for training intensity depends on many factors: the difficulty of the target language, whether the research focuses on a specific language attribute, the teaching method, and the cognitive state of the participants, among others. Kliesch et al. [44] noted that their course (4 h a day for three weeks) was too intense for their participants, leading to exhaustion and de-motivation for a majority of their participants. Older adults might be especially sensitive to language course intensity, which requires a careful choice of course intensity. However, the results of Bak et al. [46] suggest that foreign language courses with a high intensity might be more efficient in short-term modules. Especially their follow-up findings support the notion that an intensive course might be a helpful kick-off whose benefits can be maintained by practising the language at least five hours a week afterwards. Table 2 below summarizes the studies’ training methods as well as intensity and duration of the teaching designs.

### 3.5. Assessment of Language Proficiency and Language Learning Rate

Those studies with a focus on language learning rate assessed the language learning outcome with more elaborate pre- and post-test designs [42,44,45,47] than those studies that focused on the cognitive outcomes. Mackey and Sachs [42] showed that older adults are capable of significantly improving their L2 question formation as a result of a relatively short training (rpb = 0.67, *p* = 0.05). Those participants with the longest formal education, and the highest working memory spans were those who improved the best and in a more sustained manner. It is important to point out that the difference in main focus of the work done so far (either on linguistic or cognitive outcomes of foreign language training) uniquely reflects the bidirectional nature of bilingualism effect. In other words, language learning can have an effect on cognition, but cognitive differences among individuals prior to the language training may also predict linguistic success. This issue is revisited in more detail in Section 4 below. Cox’s [47] study revealed that all of her older participants were able to significantly improve their Latin skills in written interpretation (F(2, 78) = 37.01, *p* < 0.001, ηp2 = 0.49), aural interpretation (F(2, 74) = 15.49,

*p* < 0.001, ηp2 = 0.30), grammaticality judgement (F(2, 68.44) = 4.97, *p* = 0.010, ηp2 = 0.11), and written production (F(2, 68) = 25.28, *p* < 0.001, ηp2 = 0.43). After the intensive English course in the pilot study of Kliesch et al. [44], the English proficiency of all participants as measured in terms of a C-test, and an oral translation test (t(9) = 6.33, *p* < 0.0001). While the study by Pfenninger and Polz (2018) did not improve significantly in their post-tests, their results did reveal that the participants made significantly fewer mistakes in the post-tests: they produced fewer unfilled gaps and incorrect answers (Z = −2.845, punilateral = 0.002, r = −0.859), fewer orthographical errors (Z = −1.779, punilateral = 0.037, r = −0.536), and fewer phonological errors (Z = −2.937, punilateral = 0.001, r = −0.886), underscored by large effect sizes throughout. These results clearly demonstrate that older adults can benefit from a language course and possess the cognitive abilities to successfully follow a language course. It is interesting to note that in the studies that focus on the cognitive effects to ensue from a language course, language proficiency is a tool rather than a goal in itself and as such is not necessarily measured.

### 3.6. Socio-Affective Measures

Only three studies included questionnaires on socio-affective improvements in their study design (Ware et al. [43]; Kliesch et al. [44]; and Pfenninger and Polz, [45]). They either conducted semi-structured interviews, assessed the University of California Loneliness Assessment (UCLA) scale (Ware et al. [43]), handed out weekly motivation questionnaires (Kliesch et al. [44]), or compiled an exhaustive questionnaire on motivation, and social and emotional well-being (Pfenninger and Polz [45]). Especially the findings by Pfenninger and Polz underline the importance to assess the socio-affective consequences of a foreign language course for older adults. Their questionnaires revealed that all of the participants were motivated and had personal goals at the onset of the training. Following the language course, most of the participants reported to have more social contacts as a consequence of the intervention, that their self-esteem improved, and that they felt socially more recognized. These socio-affective components may crucially contribute to healthy aging and in turn to better cognitive reserve. While other activities might lead to similar positive socio-affective results, language learning might be unique as it combines the aspects of being part of a purposeful cognitively demanding activity, and which additionally has a social and communicative role. Being able to see own improvements in using a foreign language might especially strongly influence older adults’ self-esteem.

### 3.7. Cognitive Tasks and Cognitive Outcomes

The five studies focusing on cognitive improvement tested the following abilities in pre- and post-test designs: auditory attentional inhibition (Bak et al. [46]), switching (Ramos et al. [48]), general cognitive state (Ware et al. [43]), inhibition and concentration (Pfenninger and Polz [45]), and spatial intelligence, verbal intelligence, working memory, long-term associative memory, and item memory (Berggren et al. [49]). There was no overlap of cognitive tasks between those five studies, making a direct comparison more difficult. This is in line with what has been noted before as a large contributor to the mixed findings of the bilingual advantage at large (cf. van den Noort et al. [19] this special issue).

Only Bak et al. and Pfenninger and Polz found a significant improvement of cognitive abilities following the language course. Bak et al. [46] tested their participants using the Test of Everyday Attention, which consists of three tasks: the Elevator Task, the Elevator Task with Distraction, and the Elevator Task with Reversal. There was no significant improvement between pre- and post-test for the first two tasks for any group, which the researchers ascribed to the fact that the tasks were too simple for the participants (they all performed at ceiling level at the pretest already). However, the results showed that there was a general trend of improvement in the Elevator Task with Reversal between the pre- and post-test (F(2,66) = 6.65, *p* = 0.002) with a significant linear trend showing that, proportionally, the language group improved the most followed by the active control group, and then the passive control group (F(1,64) = 12.87, *p* = 0.001). There was no main effect of group (F (1,65) = 1.72, *p* = 0.195, ηp2 = 0.026), but the interaction between session and group was significant (F (1,65) = 7.15, *p* = 0.009, ηp2 = 0.099). Pairwise comparisons showed that only the language training group was able to improve the performance in the Elevator Task with Reversal significantly (t = 6.25, df = 32, *p* < 0.001, two-tailed) in comparison to the pre-test. Follow-up analyses revealed that in both the experimental and the control groups, the youngest age group (18–40 years.) scored the highest on the cognitive tasks, and the oldest age group (61–78 years.) the lowest - however, the improvement in the Elevator Task with Reversal of the experimental group was significant across all age groups. Bak et al. [46] retested 17 participants of the experimental group after nine months using the same cognitive tasks. Those subjects who continued to practise Scottish Gaelic (on average above 5 h per week) continued to show a significant improvement rate in the Elevator Task with Reversal in comparison to their first pre-test (t = 8.275, df = 7, *p* < 0.001, two-tailed), suggestive of the persistent effects of the training. Importantly, such an effect was lacking altogether for those individuals who did not continue to practise their language skills, indicating the limited timeframe of the effects. The analysis of Pfenninger and Polz [45] revealed a significant improvement in inhibition and interference as measured through a Stroop Task for both the monolingual language learners (*p* = 0.014, r = −0.897), and the bilingual language learners (*p* = 0.023, r = −0.813). However, only the monolingual group significantly improved their performance in terms of attention (*p* = 0.038, r = −0.728), whereas the bilingual group did not (*p* = 0.112, r = 0.545). Furthermore, the monolinguals outperformed the bilinguals on both cognitive tasks, which is not interpreted by the authors.

Despite their longitudinal design (Ramos et al. [48]; Ware et al. [43]), and their vast battery of assessed cognitive abilities, together with their large sample size (Berggren et al. [49]), the other three studies were not able to detect any improvements in the cognitive abilities that were assessed. Ramos et al. [48] report nearly identical performance in the Colour-Shape Switching Task by both their active language learning condition and passive control participant groups after months of intervention. Ware et al. [43] could not find a significant difference in the Montreal Cognitive Assessment, a task designed to assess the general cognitive state of older adults, either. This could be explained by the “study’s small sample size, as well as participants’ generally high cognitive level.” (p. 7). Berggren et al.’s study could not detect any cognitive-enhancing effects of foreign language learning in their older adults either, leading to their conclusion that foreign language learning does not incur positive effects on general cognitive functioning. However, it should be noted that Berggren et al.’s language course goal appears to have been solely lexical acquisition, as language progress was assessed using vocabulary tests only and with that perhaps not training a wide array of cognitive skills. In addition, each of the pre- and post-test assessments consisted of two sessions of 3.5 h each (a total of 7 h per battery testing) that were assessed in groups, very likely leading to fatigue playing role. Nonetheless, this study relates to whether language learning indeed does enhance general cognitive functioning in older adults, which should be investigated in future studies. Table 3 below details the cognitive outcomes of the language interventions, as found by the studies investigated.

## 4. Future Directions: Cognitive, Social and Linguistic Effects of and on Third Age Language Learning

Within this spectrum of experiences, bilingualism has taken a prominent and yet complex role. In a field characterized by mixed findings, the current trend to focus on individual factors that modulate the bilingual advantage [19] as well as a focus on a monolingualism to bilingualism continuum rather than divide (cf. Reference [25]) is a very important one. But while such approaches go a long way towards explaining mixed findings, they do not provide a good answer to the question of what constitutes language and cognitive control and the transfer between them (cf. Reference [51]). We have posited older adulthood as a testing ground to elucidate this very issue; first of all, because cognitive and social dimensions change as a function of age, causing effects ensuing from (lifelong) bilingualism to surface more prominently (although necessarily stronger) at the upper end of the lifespan. At the same time, individual lifetime experiences interact more intricately with bilingualism at this life stage, making it harder to disentangle bilingualism effects, as shown in the first part of this paper. Crucially, however, the brain remains much more flexible in learning new skills than previously assumed (cf. Reference [36]) and teaching a new language to older adults is the most recent addition in a field looking at late-life interventions to improve cognition and well being. Not only can bilingualism effects thus be introduced in a much more controlled setting where its effects can be measured and set off against other types of interventions, but individual differences in cognitive, social and language abilities of older adults themselves can be used as predictors of language learning success. In other words, focusing on third-age language learning highlights the bidirectional in bilingual experiences. The meta-study that formed the second part of this paper showed this potential through 8 studies conducted in the emergent field of third-age language learning so far. While the variability of these past investigations (most notably in some of them investigating the cognitive and social outcomes of language learning versus others targeting the language outcomes themselves) reflects this bi-directionality, future studies in this domain would do well to build on a number of principles that themselves are rooted in what past work about bilingual experiences has taught us, as captured in Section 2 and Section 3 above:

### 4.1. Balancing Language, Cognition and Social Dimensions

The work done so far shows a clear divide: third-age language learning is often used as a tool to promote healthy aging (with a focus on cognitive enhancements), where the linguistic outcomes play a secondary role or no role at all. Alternatively, studies assess how the attainment and rate of L2 learning in older adulthood may or may not include cognitive and social indices as predictors of this success. Future work should ideally combine all three facets. Especially in older adulthood the three cannot be seen in isolation. Especially in longitudinal designs, cognitive, social and linguistic dimensions can be dynamically used as both dependent and independent variables, ideally using a battery of tests validated at the older end of the life spectrum.

### 4.2. Intensity and Type of the Language Intervention

Most training studies so far use a standard classroom setting, with only Bak et al. [46] adding exposure through Gaelic entertainment in the evenings in addition to formal classroom instruction to the design. Two future directions should be outlined here: the type of classroom instruction should first of all be assessed more thoroughly in terms of benefits for older adults (e.g., implicit vs. explicit instruction, computer-based vs. class-room setting, group learning vs. one-on-one interaction). The focus of instruction should also be examined: null-findings surfaced most in past work which focused mainly on lexical acquisition, perhaps as this was not challenging enough. As a second goal for future studies, out of class exposure should also be examined. For younger adults, (over)hearing a language in the environment significant aided subsequent classroom instruction of that language [52]. How this plays a role in older adulthood remains an empirical question.

### 4.3. The Issue Of Thresholds

Related to the previous point, it has been coined that language training regimes should last at least 3 months for cognitive and social effects to be manifested [36], although seniors were not specifically targeted. Due to general cognitive slowing, it likely takes longer for effects to emerge at the upper end of the lifespan, but how much intervention and/or exposure is enough to observe effects and detailing which effects are expected at which timeframe is very much needed. Likewise, Bak et al. [46] found that any cognitive effects returned to baseline in the absence of continued practice. In his recent Dynamic Restructuring Model (or DRM), Pliatsikas [53] captures the variability in experience-based neuroplasticity—including on the basis of evidence in seniors—and shows that brains are flexible in adapting to increased demands such as those that ensue from language training but such effects can disappear when the training ceases. This idea of what constitutes a critical mass of threshold in the context of foreign language training is not new (cf. Reference [54]). Likewise, work in relation to the savings account (cf. reviewed in Reference [55]) has traditionally examined which portions of languages once learned are resilient to attrition despite potentially years of non-use. This can be extended to examine whether relearning an old language (so essentially relearning) brings about differential cognitive, social and linguistic effects than learning an entirely new language in older adulthood.

### 4.4. Design and Method

With much having been said already about designs, some future avenues still need to be addressed. In the third-age language studies done so far, some have included active and/or passive control conditions while this was absent in others, dependent on their research aims. To disentangle language effects from other influences and to thus shed light on language versus cognitive control, we advocate a standard inclusion of both active and passive control groups. The studies detailed above already show the variety of active control options. While this choice is irrelevant in some respects, it is recommended that the control activities are mastery as coming to terms with a new language and ideally have themselves been associated with cognitive reserve in older adulthood. Examples include university of the third age courses, or musical training. In terms of impact, while the available small-scale studies having been instrumental in exploring the linguistic, cognitive and social effects of third-age language learning, the next step is upscaling, with work not only targeting healthy adults but also geriatric patients, most notably those suffering from memory and/or mood complaints. It is in this non-healthy seniors that language, cognition and social dimensions are most intricately linked [56], allowing a unique view on how language and cognitive control in seniors are related.

Gerontology, the scientific study of aging, is a multifaceted field, informed by disciplines such as biology, psychology, and sociology. (Bilingual) language use has not featured prominently in this domain, but given its position as both a pertinent life experience as well as its potential as an intervention tool to promote healthy aging through third-age language learning, we argue that languages should be a standard addition to the broader gerontology field. Third-age language learning itself brings together the disciplines of education, applied linguistics, neuroscience, psychology and sociology where the sum of all these fields. And, with time, the bidirectional in bilingual, is perhaps more accurately captured in the multidirectional in multilingual experiences.

## Figures and Tables

**Table 1:** Participant setup and general research questions of the included studies.

Study	Participants	Groups	Study Aims and Scope
	n, (Gender), Age in Years		
Mackey & Sachs (2012)	9 (4 m./5 f.) 65–89 (mean: 72)	no control group	Mackey & Sachs investigated whether verbal working memory, and phonological short-term memory predict improvement in L2 question formation of Spanish L1 older adults with different lengths of residency in the USA. The older adults participated English question formation in five training sessions spread over five weeks with a train ed L1 speaker and with a focus on interaction and feedback.
Bak et al. (2016)	77 (31 m./46 f.) 18–78 (mean: 49.2)	language learning group, active control group, passive control group	Bak et al. investigated how a 1-week intensive foreign language course in Scottish Gaelic, consisting of a total of 19.5 h of language teaching, changed the performance in auditory tests of attentional inhibition. They compared their participants across age groups (young adults, middle-aged, and older adults), and also compared the experimental group to active and passive control groups. In addition, they followed half of their experimental group and re-tested them nine months after the intensive language course with the same cognitive task battery.
Cox (2017)	43 (16 m./27 f.) 60–82 (mean: 68.87)	mono-/bilingual with/without explicit instruction	Cox investigated the influence of late Spanish bilingualism and explicit grammar instruction on the acquisition of basic morpho-syntax of Latin in English L1 speakers. The older adults underwent a short computer-based training of basic Latin in two sessions followed by post-test Latin assessments.
Ramos et al. (2017)	43 (22 m./21 f.) 60–80 (mean: 68.3)	language learning group, passive control group	Ramos et al. investigated the influence of a long-term (eight month) class-taught language course of Basque on the cognitive ability of switching in Spanish monolingual older adults.
Ware et al. (2017)	14 (5 m./9 f.) 63–90 (mean: 75.42)	no control group	Ware et al. focused on the effect of a 4-month foreign language course of English on the general cognitive state and well-being of French older adults with different proficiency levels of English.
Kliesch et al. (2018)	10 (6 m./4 f.) 65–74 (mean: 68.2)	no control group	Kliesch et al.’s pilot study examined which cognitive abilities and other factors (such as motivation, or time spent on self-study) best predict the learning rate of a foreign language in older adults. Their subjects were monolingual speakers of Austrian German and they underwent an intensive class-taught course of English.
Pfenninger & Polz (2018)	12 (4 m./ 8 f.) 63–89 (mean: 71.83)	mono-/bilingual	Pfenninger & Polz’s pilot study looked into the effects of an intensive English course on the abilities of inhibition, concentration and overall well-being of Austrian older adults. Furthermore, this study compared German monolinguals to sequential Slovenian-German bilinguals in order to investigate the effect of prior bilingualism.
Berggren et al. (2018)	160 (60 m./100 f.) 65–75 (mean: 69.35)	language learning group and active control group	Berggren et al. conducted a large-scale study investigating the influence of an 11-week foreign language course of Italian on general cognitive abilities of monolingual Swedish older adults. The study included an active control group undergoing yoga relaxation classes.

**Table 2:** Language training method, intensity, and duration.

Study	Language Training Method	Training Duration
Mackey & Sachs (2012)	communication sessions with trained L1 speakers	non-intensive & short
		(5 sessions during 5 weeks)
		total: estimated <10 h
Bak et al. (2016)	classes taught by trained teachers	intensive & short
		total: 19.5 h (in 1 week)
Cox (2017)	computer-based learning of	non-intensive & short
	basic language features	(2 sessions within a week)
		total: estimated <10 h
Ramos et al. (2017)	classes taught by trained teachers	semi-intensive & long
		(3.5 h/week for 8 months)
		total: estimated 110 h
Ware et al. (2017)	course with teacher & technology supported	semi-intensive & long
		(2 h/week for 4 months)
		total: 16 h
Kliesch et al. (2018)	classes taught by trained teachers	intensive & semi-long
		(4 h/day for 3 weeks)
		total: 60 h
Pfenninger & Polz (2018)	classes taught by trained teachers	semi-intensive & short
		(6 h/week for 4 weeks)
		total: 24 h
Berggren et al. (2018)	classes taught by trained teachers	semi-intensive & semi-long
		(5 h/week for 11 weeks)
		total: 55 h

**Table 3:** Cognitive tasks and outcomes.

Study	Cognitive Tests	Language Training as Cognitive Boost?
Mackey & Sachs (2012)	verbal working memory (listening-span (LS) task) & phonological short-term memory (PSTM, non-word recall test)	not tested
Bak et al. (2016)	Test of Everyday Attention different aspects of attention	yes, with significance (only for Elevator task with Reversal)
Cox (2017)	Digit-Symbol Coding Task (processing speed under low cognitive demands)	not investigated
Ramos et al. (2017)	Colour-Shape Switching Task	not significant
Ware et al. (2017)	French version of the Montreal Cognitive Assessment (MoCA), brief cognitive test of global cognitive functioning in older adults	not significant
Kliesch et al. (2018)	a battery of nine standardized tests such as Stroop Task, Eriksen Flanker Task or Reading Span Task in order to assess the cognitive skills of inhibition, shifting, working memory, delayed recall, and verbal fluency	not tested
Pfenninger & Polz (2018)	Stroop Task (verbal & non-verbal inhibition skills), concentration test for geriatric patients (Alters-Konzentrations-Test A-K-T, Gatterer, 1989; attention & concentration)	partially yes (Stroop task), with significance
Berggren et al. (2018)	test battery of 10 items: 2 tests of spatial intelligence (Ravens matrices (Raven, 1960) & WASI-II Matrix Task (Wechsler, 1999)); 3 tests of verbal intelligence (Analogies, Syllogisms, and Verbal Inference; Ekstrom et al., 1976); 2 tests of working memory (Numerical updating, n-back); 3 tests of long-term associative memory & item memory using different types of stimuli (word-word, face-name, picture-picture)	not significant

## Abbreviations

The following abbreviations are used in this manuscript:

MDPI and Multidisciplinary Digital Publishing Institute
DOAJ and Directory of open access journals
